# Invasive Methicillin-Resistant *Staphylococcus aureus* Infections Among Persons Who Inject Drugs — Six Sites, 2005–2016

**DOI:** 10.15585/mmwr.mm6722a2

**Published:** 2018-06-08

**Authors:** Kelly A. Jackson, Michele K. Bohm, John T. Brooks, Alice Asher, Joelle Nadle, Wendy M. Bamberg, Sue Petit, Susan M. Ray, Lee H. Harrison, Ruth Lynfield, Ghinwa Dumyati, William Schaffner, John M. Townes, Isaac See

**Affiliations:** ^1^Division of Healthcare Quality Promotion, National Center for Emerging and Zoonotic Infectious Diseases, CDC; ^2^Division of Unintentional Injury Prevention, National Center for Injury Prevention and Control, CDC; ^3^Division of HIV/AIDS Prevention, National Center for HIV/AIDS, Hepatitis, STD, and TB Prevention, CDC; ^4^Division of Viral Hepatitis, National Center for HIV/AIDS, Hepatitis, STD, and TB Prevention, CDC; ^5^California Emerging Infections Program, Oakland, California; ^6^Colorado Department of Public Health and Environment, Denver, Colorado; ^7^Connecticut Department of Health, Hartford, Connecticut; ^8^Georgia Emerging Infections Program and the Atlanta Veterans Affairs Medical Center, Decatur, Georgia; ^9^Maryland Emerging Infections Program and Johns Hopkins Bloomberg School of Public Health, Baltimore, Maryland; ^10^Minnesota Department of Health, St. Paul, Minnesota; ^11^New York-Rochester Emerging Infections Program and University of Rochester, Rochester, New York; ^12^Vanderbilt University School of Medicine, Nashville, Tennessee; ^13^Oregon Health & Science University, Portland, Oregon.

In the United States, age-adjusted opioid overdose death rates increased by >200% during 1999–2015, and heroin overdose death rates increased nearly 300% during 2011–2015 (*1*). During 2011–2013, the rate of heroin use within the past year among U.S. residents aged ≥12 years increased 62.5% overall and 114.3% among non-Hispanic whites, compared with 2002–2004 (*2*). Increases in human immunodeficiency virus (HIV) and hepatitis C virus (HCV) infections related to increases in injection drug use have been recently highlighted (*3*,*4*); likewise, invasive bacterial infections, including endocarditis, osteomyelitis, and skin and soft tissue infections, have increased in areas where the opioid epidemic is expanding (*5*–*7*). To assess the effects of the opioid epidemic on invasive methicillin-resistant *Staphylococcus aureus* (MRSA) infections during 2005–2016, surveillance data from CDC’s Emerging Infections Program (EIP) were analyzed (*8*). Persons who inject drugs were estimated to be 16.3 times more likely to develop invasive MRSA infections than others. The proportion of invasive MRSA cases that occurred among persons who inject drugs increased from 4.1% in 2011 to 9.2% in 2016. Infection types were frequently those associated with nonsterile injection drug use. Continued increases in nonsterile injection drug use are likely to result in increases in invasive MRSA infections, underscoring the importance of public health measures to curb the opioid epidemic.

Active, population-, and laboratory-based surveillance data collected through the Healthcare-Associated Infections/Community Interface (HAIC) component of CDC’s EIP during 2005–2016 were analyzed to assess the effects of the opioid epidemic on invasive MRSA infection. A case was defined as the isolation of MRSA from a normally sterile site (e.g., blood, cerebrospinal fluid, or bone) from a surveillance area resident. National invasive MRSA disease prevalence (adjusted for age, race, sex, and dialysis) among persons aged ≥13 years who inject drugs and among persons aged ≥13 years who do not inject drugs were estimated for 2011 from EIP/HAIC data using a previously described method (*8*); invasive MRSA rates per 100,000 persons in both groups (and the corresponding rate ratio) were calculated in conjunction with a published population point estimate of the U.S. population aged ≥13 years who injected drugs in the previous year for 2011 (*9*). The six-site surveillance area used for the remainder of this report included California (three counties); Connecticut (statewide); Georgia (eight counties); and Minnesota, New York, and Tennessee (one county each). Demographic characteristics and clinical diagnoses of invasive MRSA cases among persons who inject drugs were compared with those among persons who do not inject drugs. The proportion of invasive MRSA cases that occurred among persons who injected drugs (among all invasive MRSA cases) was calculated overall and by site for each year; significance of trends was analyzed using linear regression. P values <0.05 were considered statistically significant.

Among invasive MRSA cases occurring in persons who inject drugs, demographics and health care–associated risk factors for cases ascertained during 2005–2010 were compared with those that occurred during 2011–2016 to describe changes over time. Health care–associated risk factors include specimen collection for culture >3 days after hospital admission; dialysis, hospitalization, surgery, or long-term care residency in the 12 months preceding culture; and/or presence of a central venous catheter ≤2 days before invasive MRSA culture collection. Cases among persons with none of these risk factors were considered community-associated. Trends in the proportion of invasive MRSA cases that occurred among persons who inject drugs also were assessed in three sites that reported data from 2005–2014 only (Colorado and Maryland [one county each]; Oregon [three counties]).

Among 39,050 invasive MRSA cases reported from six sites during 2005–2016, a total of 2,093 (5.4%) occurred in persons who injected drugs. The estimated rate of invasive MRSA among persons aged ≥13 years who injected drugs in the previous year was 472.2 per 100,000 in 2011, and the estimated rate among persons aged ≥13 who did not inject drugs in the previous year was 29.0 per 100,000 (rate ratio [RR] = 16.3; 95% confidence interval [CI] = 15.7–16.8). Overall, cases of invasive MRSA among persons who inject drugs were more likely than cases among persons who did not inject drugs to occur in persons who were younger (median age = 45 versus 63 years; p<0.05) and to be community-associated infections (odds ratio [OR] = 4.4, 95% CI = 4.0–4.8). Clinical diagnoses frequently associated with injection drug use were more common among patients with invasive MRSA who injected drugs than among those who did not (Table), including septic embolism, endocarditis, abscess (skin and internal), cellulitis, and osteomyelitis.

**TABLE:** Clinical diagnoses of cases of invasive methicillin-resistant *Staphylococcus aureus* (MRSA) infection, by injection drug use status — Emerging Infections Program, six surveillance sites,* 2005–2016

Infection type^†^	Cases among persons who inject drugs (n = 2,093), no. (%)	Cases among persons who do not inject drugs (n = 36,957), no. (%)	OR (95% CI)
Septic emboli^§^	208 (14.9%)	340 (1.4%)	12.7 (10.6–15.2)
Endocarditis	426 (20.4%)	1,601 (4.3%)	5.6 (5.0–6.3)
Abscess (not skin)	350 (16.7%)	1,920 (5.2%)	3.7 (3.2–4.1)
Skin abscess^¶^	204 (12.8%)	1,361 (4.7%)	3.0 (2.5–3.5)
Meningitis	243 (11.6%)	169 (0.5%)	2.5 (1.6–3.9)
Septic arthritis	240 (11.4%)	2,186 (5.9%)	2.1 (1.8–2.4)
Cellulitis	367 (17.5%)	3,459 (9.4%)	2.1 (1.8–2.3)
Traumatic wound infection	25 (1.2%)	254 (0.7%)	1.7 (1.2–2.6)
Empyema	60 (2.9%)	650 (1.8%)	1.6 (1.3–2.2)
Osteomyelitis	337 (16.0%)	4,073 (11.0%)	1.5 (1.4–1.7)
Pneumonia	282 (13.5%)	4,655 (12.6%)	1.1 (0.9–1.2)
Bacteremia	1,541 (73.6%)	28,073 (76.0%)	0.9 (0.8–0.98)
Septic shock	132 (6.3%)	2,799 (7.6%)	0.8 (0.7–1.0)
Bursitis	23 (1.1%)	717 (1.9%)	0.6 (0.4–0.9)
Decubitus/Pressure ulcer infection	28 (1.3%)	974 (2.6%)	0.5 (0.3–0.7)
Internal surgical site infection	22 (1.1%)	821 (2.2%)	0.5 (0.3–0.7)
Urinary tract infection	55 (2.6%)	2,348 (6.4%)	0.4 (0.3–0.5)
Surgical incision infection	22 (1.1%)	1,124 (3.0%)	0.3 (0.2–0.5)
Peritonitis	8 (0.4%)	538 (1.5%)	0.3 (0.1–0.5)
Arteriovenous fistula/Graft infection**	7 (0.6%)	447 (2.1%)	0.3 (0.1–0.6)
Catheter site infection**	12 (1.0%)	686 (3.2%)	0.3 (0.2–0.5)
Chronic ulcer/Wound infection^††^	14 (1.4%)	709 (4.0%)	0.3 (0.2–0.6)

**Abbreviations:** CI = confidence interval; OR = odds ratio.

* California (three counties), Connecticut (statewide), Georgia (eight counties), Minnesota (one county), New York (one county), and Tennessee (one county).

^†^ Cases can have more than one infection type.

^§^ Variable added to surveillance in 2008. For persons who inject drugs n = 1,395; for persons who do not inject drugs n = 24,987.

^¶^ Variable added to surveillance in 2007. For persons who inject drugs n = 1,589; for persons who do not inject drugs n = 28,860.

** Variable added to surveillance in 2009. For persons who inject drugs n = 1,201; for persons who do not inject drugs n = 21,334.

^††^ Variable added to surveillance in 2010. For persons who inject drugs n = 1,039; for persons who do not inject drugs n = 17,668.

The proportion of invasive MRSA cases that occurred among persons who inject drugs approximately doubled in some sites (counties in Connecticut, Georgia, Minnesota, and Tennessee) after 2011. In the six-site catchment area, the percentage of invasive MRSA cases among persons who inject drugs declined from 6.4% in 2005 to 3.5% in 2010 (p<0.05), but subsequently increased steadily to 9.2% in 2016 (p<0.05) (Figure). Among invasive MRSA cases that occurred among persons who inject drugs, cases during 2011–2016 were more likely to occur in persons who were white (OR = 1.7; 95% CI = 1.4–2.0) and be community-associated (OR = 1.3; 95% CI = 1.1–1.6) than were cases during 2005–2010. In two of three sites (Colorado and Oregon) that reported data from 2005–2014 only, similar increases in the proportion of invasive MRSA cases that occurred among persons who inject drugs after an initial decrease (2005: 11.1% of cases; 2011: 10.6%; 2014: 15.2%) were observed.

**FIGURE:**
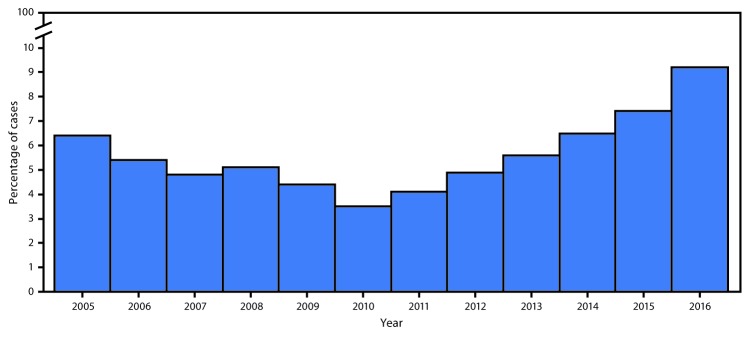
Percentage of invasive methicillin-resistant *Staphylococcus aureus* cases among persons who inject drugs, by year — Emerging Infections Program, six surveillance sites,* 2005–2016 * California (three counties), Connecticut (statewide), Georgia (eight counties), Minnesota (one county), New York (one county), and Tennessee (one county).

## Discussion

In six sites, invasive MRSA infections disproportionately affected persons who inject drugs. In this analysis, invasive MRSA infections that occurred among persons who inject drugs were those frequently associated with nonsterile injection drug use; demographic shifts in the population of invasive MRSA infections among injection drug users mirror those observed in the ongoing opioid epidemic, such as the increased proportion of cases among whites. A decline and subsequent rise in the proportion of invasive MRSA cases among persons who inject drugs was observed in the six-site catchment area during 2005–2016 and in two additional sites for which data were available through 2014; similar patterns were seen in the incidence of acute HCV* and in the rate of drug overdose deaths involving heroin (*1*), with notable increases in both beginning around 2010.

The findings in this report are subject to at least five limitations. First, injection drug use status in medical records is possibly misclassified, which could result in an under- or overestimation of the percentage of MRSA infections in injection drug users. Second, rates were based on national estimates of both invasive MRSA case counts and the population of persons who inject drugs that might not be accurate. Third, the rates are based on 2011 data because this is the only year for which population estimates for the number of persons who inject drugs is available. This might be an underestimate if current injection drug use practices are higher risk. Fourth, site-specific counts of persons who inject drugs were not available, precluding the calculation of site-specific rates. Finally, invasive methicillin-sensitive *Staphylococcus aureus* surveillance began in 2016 and could not be included in this report to describe the impact of the opioid epidemic on these infections.

Although much attention has focused on the transmission of blood-borne pathogens such as HIV and hepatitis B and C viruses related to injection drug use, infections from skin flora such as *Staphylococcus aureus* are also important and might not be prevented solely by programs focused on preventing blood-borne pathogen transmission. Increases in nonsterile injection drug use are likely to result in increases in the occurrence of invasive MRSA infections among persons who inject drugs, underscoring the importance of public health measures to curb the opioid epidemic. Effective interventions include primary prevention of opioid misuse through guideline-concordant opioid prescribing; treatment of opioid use disorder with medication-assisted therapies; community-based comprehensive syringe services programs that provide access to sterile equipment used to inject drugs and its safe disposal; and education on safer injection practices, wound care, and early warning signs of serious infections associated with injection drug use.

**Summary**

**What is already known about this topic?**

The ongoing opioid epidemic is associated with increases in human immunodeficiency virus and hepatitis C infections and infection syndromes such as endocarditis.

**What is added by this report?**

Persons who inject drugs were an estimated 16.3 times more likely to develop invasive methicillin-resistant *Staphylococcus aureus* (MRSA) infections than others. Invasive MRSA from injecting drugs increased from 4.1% of invasive MRSA cases to 9.2% (2011–2016).

**What are the implications for public health practice?**

Increases in nonsterile injection drug use can cause increases in MRSA infections, underscoring the importance of public health interventions, including prevention of opioid misuse, providing medication-assisted treatment, syringe services programs, and education on safer injection practices to prevent infections from skin flora.

## Footnotes

*
https://www.cdc.gov/hepatitis/statistics/2015surveillance/commentary.htm .
